# iRECIST: how to do it

**DOI:** 10.1186/s40644-019-0281-x

**Published:** 2020-01-03

**Authors:** Thorsten Persigehl, Simon Lennartz, Lawrence H. Schwartz

**Affiliations:** 1 Department of Diagnostic and Interventional Radiology, Faculty of Medicine and University Hospital Cologne, University Cologne, Kerpener Straße 62, 50937 Cologne, Germany; 2 Else Kröner Forschungskolleg Clonal Evolution in Cancer, University Hospital Cologne, Weyertal 115b, 50931 Cologne, Germany; 3 Department of Radiology, New York Presbyterian Hospital, Columbia University Irving Medical Center, New York, NY 10032 USA

**Keywords:** iRECIST, Immunotherapy, Therapy monitoring, Response evaluation, Pseudoprogression

## Abstract

**Background:**

iRECIST for the objective monitoring of immunotherapies was published by the official RECIST working group in 2017.

**Main body:**

Immune-checkpoint inhibitors represent one of the most important therapy advancements in modern oncology. They are currently used for treatment of multiple malignant diseases especially at advanced, metastatic stages which were poorly therapeutically accessible in the past. Promising results of recent studies suggest that their application will further grow in the near future, particularly when used in combination with chemotherapy. A challenging aspect of these immunotherapies is that they may show atypical therapy response patterns such as pseudoprogression and demonstrate a different imaging spectrum of adverse reactions, both of which are crucial for radiologists to understand. In 2017 the RECIST working group published a modified set of response criteria, iRECIST, for immunotherapy, based on RECIST 1.1 which was developed for cytotoxic therapies and adapted for targeted agents.

**Conclusion:**

This article provides guidance for response assessment of oncologic patients under immunotherapy based on iRECIST criteria.

## Background

Immune-checkpoint inhibitors have become an integral part of many cancer therapy regimens [1] and their importance continues to grow as numerous immunotherapeutic agents are put into active preclinical development and clinical trials. Most of the clinically approved immunotherapeutic agents are based on modulation of T-cell activation either by a therapeutic blockade of cytotoxic T-lymphocyte antigen 4 (CTLA-4), programmed death 1 receptor (PD-1), or programmed death ligand 1 (PD-L1) [2, 3].

Positive therapeutic effects of immunotherapy has been demonstrated in the treatment of malignant melanoma, renal cell carcinoma, Hodgkin lymphoma, non-small cell lung cancer (NSCLC), squamous cell carcinoma of the head and neck, colon carcinoma, ovarian carcinoma, and urothelial carcinoma, partially resulting in a substantial improvement in patient survival [4–9]. Despite a strong and positive therapeutic effect, immune-checkpoint inhibitors may demonstrate atypical response patterns, such as delayed tumor size reduction, mixed response, or an initial tumor burden increase due to an increase in lesion size and/or occurrence of newly detectable tumor lesions with subsequent decrease in tumor burden, the so-called pseudoprogression [10]. Additionally, hyperprogression following immunotherapy initialization has been described as a ≥ 2-fold increase in tumor growth kinetic as compared to pretherapeutic state [11, 12]. Furthermore, immune-related adverse events such as immunotherapy-associated pneumonitis, colitis, hypohysitis, thyroiditis, pancreatitis, and arthritis, could be observed during various immunotherapies [13, 14].

**Fig. 1:**
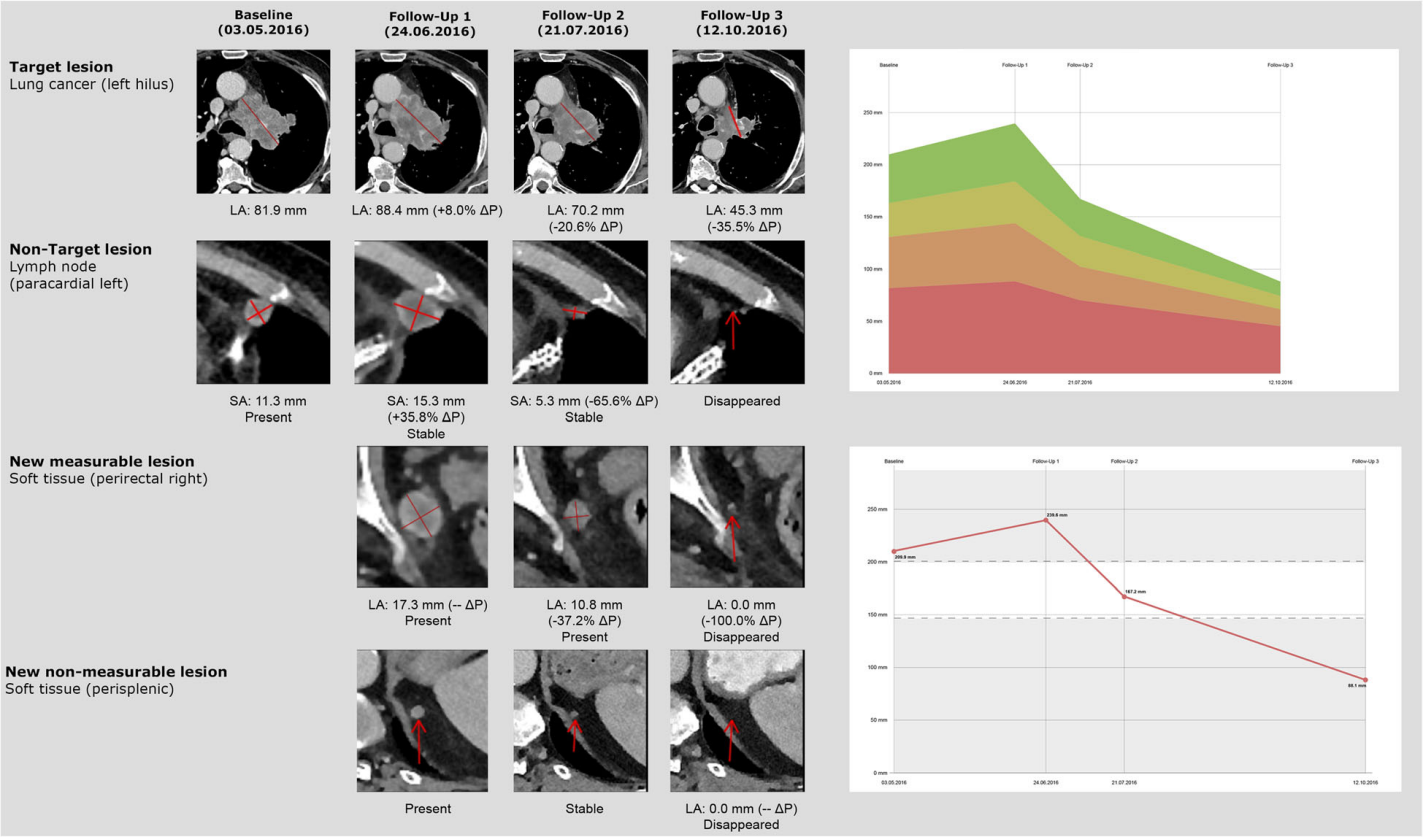
Example of pseudoprogression in a patient with metastatic lung cancer. Target lesion: after initial increase of the lung cancer the lesion showed a subsequent shrinkage. Non-target lesion: initial increase of a paracardial lymph node. New measureable lesion: at the first follow-up new perirectal soft tissue lesion (17 mm) which decreased at the following examinations. New non measureable lesion: further small new perisplenic lesion (9 mm) which disappeared completely after 4 month

The frequency of pseudoprogression as well as immune-related adverse events are quite variable, depending on the primary disease site, the specific immunotherapy agent and the use of drug combinations. In an article by Wolchok et al., it was revealed that pseudoprogression in malignant melanoma under Ipilimumab (anti-CTLA-4) with subsequent therapy responses occurring in about 13% of progressive patients [15]. Hodi et al. reported pseudoprogression with Nivolumab (anti-PD-1) treatment in about 8% of the patients examined [16]. With regards to Pembrolizumab (anti-PD-1), Hodi et al. demonstrated that patients with advanced malignant melanoma showed an early pseudoprogression (≥25% increase in tumor burden in week 12, not confirmed as progressive disease at subsequent follow-up) in about 5% and a late pseudoprogression in about 3% of the cases (≥25% increase in tumor burden at any imaging assessment after week 12, not confirmed as progressive disease in subsequent follow-up), equaling a total pseudoprogression rate of about 7%. As compared to melanoma, data on pseudoprogression for other tumor entities are sparse, yet indicate lower pseudoprogression rates, e.g. for non-small-cell lung cancer (NSCLC) pseudoprogression rates were reported to account for 0–3.2% of progressions [7, 17, 18], while for renal cell carcinoma and bladder cancer, they were reported to be only about 1.8 and 1.5%, respectively [19, 20]. Similarly, the pseudoprogression rate for squamous cell carcinoma of the head and neck was reported to be around 2% [8]. However, all these data demonstrate that an increase in tumor size, is more likely to be true tumor progression rather than pseudoprogression. However, some patients with real pseudoprogression will have an overall outcome benefit by continuing the immunotherapy (Fig. 1).

The radiological response assessment of classic cytostatic and cytotoxic tumor therapies with the ‘Response Evaluation Criteria in Solid Tumors’ (RECIST 1.1) have been successfully validated in numerous clinical studies and thus RECIST 1.1 represent the most frequent currently applied response criteria in solid tumors [21, 22]. Regarding the assessment of therapy responses under immunotherapy, it was however shown that the atypical response patterns in some cases may lead to incorrect determination of the response status. In the case of a measurable lesion increase or detection of a previously occult tumor lesion, RECIST 1.1 would fail to recognize the potential pseudoprogression and long-term effectiveness of immunotherapy. Since significant tumor growth and/or newly detectable tumor lesions will generally be classified as progressive disease (PD) based on RECIST 1.1, this could result in an erroneous termination of the treatment and unjustified patient exclusion from clinical studies.

### iRECIST criteria

To address this limitation of RECIST 1.1 in cases of pseudoprogression under immunotherapy, Wolchok et al. developed modified ‘immune-related Response Criteria’ (irRC) based on the WHO criteria for the first time in 2009 [15]. In 2013 and 2014, bi-dimensional irRC were adapted to the uni-dimensional irRECIST (immune-related RECIST) criteria [23, 24]. According to irRC and irRECIST, new measurable tumor lesions are to be added to the sum of the target lesions, while only a significant increase (irRC ≥25%; irRECIST ≥20%) results in determination of tumor progression (iPD = ‘immune-related progressive disease’). One point of criticism with respect to these criteria, particularly irRC, was that non-measurable tumor lesions (i.e. non-target lesions) did not contribute to tumor progression. Moreover, in case of stable or only a minor size decreases following pseudoprogression, iPD was confirmed according to irRC and irRECIST. In the following years, various interpretations of irRC and irRECIST have been proposed, leading to much inconsistency between different studies depending on which response assessment protocol was utilized. To address this issue, the official RECIST Working Group (http://www.eortc.org/recist) published the new iRECIST guideline in 2017 [25] for assessing response to immunotherapy in clinical trials.

### iRECIST – how to do it

The basic principles of defining tumor lesions as measurable or non-measurable and assessing tumor responses used in iRECIST remain unchanged from RECIST 1.1. The most important change is in the introduction of an additional follow-up to confirm or withdraw an ‘unconfirmed’ tumor progression after initial increase in size. Similar to RECIST 1.1, iRECIST is primarily based on the use of computed tomography (CT) and magnetic resonance imaging (MRI), while inclusion of clinically visible superficial lesions in malignant melanoma is possible as well [19]. Contrast-enhanced CT or MRI examinations with a slice thickness of ≤5 mm are preferred in order to achieve a high degree of reproducibility. Transversal (axial) orientation might be preferred due to a higher reproducibility during subsequent follow-up examinations, but sagittal or coronal orientation might be favored for some tumor locations, e.g. metastases in the spinal cord. However, the identical slice orientation must be kept during subsequent follow-up. In general, soft tissue lesions should be preferred measured in the soft tissue window and pulmonary lesions in the lung tissue window. However, in some cases measurement of lung lesions in the soft tissue window might be preferential, e.g. in the presence of adjacent pulmonary vessels or atelectasis. The sole use of sonography or a ‘low-dose’ FDG-PET/CT without contrast-enhanced acquisitions is not permitted. Functional imaging information, such as the FDG positivity of lesions, can be additionally considered within RECIST 1.1 to support the determination of a complete response (iCR) or of progressive disease (iPD), but metabolic response classification is not conducted [26].

### Baseline evaluation

**Fig. 2:**
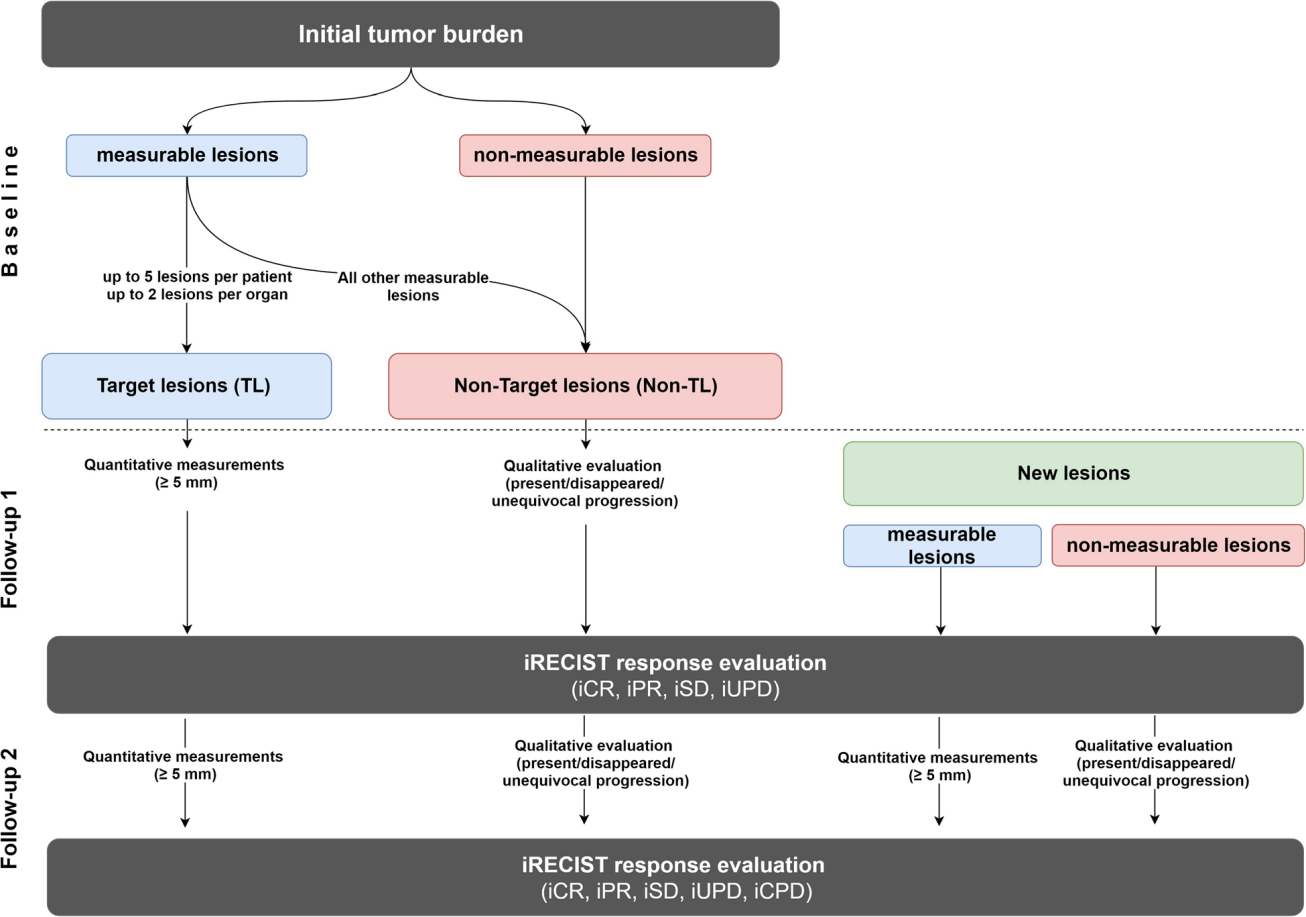
Schematic overview on baseline and follow-up assessment according to iRECIST

The baseline examination is supposed to be done as close to the start of immunotherapy as possible; in most studies, the longest acceptable interval between baseline scan and therapy start is 4 weeks. At baseline, iRECIST is used similarly to RECIST 1.1 to determine the total tumor burden by defining target and non-target lesions. For that purpose, a distinction is made between measurable and non-measurable target lesions (TL) and non-target lesions (Non-TL) (Fig. 2) [13].

In principle, all measurable solid tumor manifestations with a minimum long axis diameter (LAD) ≥ 10 mm (or at least double slice thickness), nodal lesions with a short axis diameter (SAD) ≥ 15 mm and clinical measurements of superficially localized tumor lesions ≥10 mm (documented photographically using a tape measure) can be defined as target lesions. Of these potential target lesions, analogous to RECIST 1.1, up to 5 lesions per patient, can be determined within iRECIST, of which a maximum of 2 lesions per organ can be defined as target lesions. Paired organs, such as lung or kidneys, and organ systems, such as the skeletal or lymphonodal systems, are understood as an organ group for which a maximum of 2 target lesions can be defined. The individual quantitative measurement results of the selected target lesions are noted and documented as a baseline target sum. This baseline sum diameters are used as reference to further characterize any objective tumor regression or progression in the measurable dimension of the disease.

Non-target lesions are lesions that may not be measured with a sufficient amount of reproducibility, e.g. solid tumor lesions < 10 mm, lymph node metastases with a SAD ranging between 10 and 14 mm and tumor manifestations without clear borders like infiltrative organ metastases, lymphangitis carcinomatosa, or lesions with highly variable distribution patterns, such as malignant pleural and pericardial effusion or ascites. In addition to these Non-TL, all other potential measureable target lesions which have not been selected for the category TL are also added to the Non-TL category. Several tumor lesions of one organ could be combined into one organ group, such as ‘multiple lung metastases’ or ‘diffuse liver metastasis’. Non-TL are qualitatively documented as ‘present’ and do not require a specific indication of quantitative size or absolute number. This procedure is intended to warrant complete lesion documentation in case of uncountable metastases.

According to RECIST 1.1, there are specific recommendations regarding bone lesions, cystic lesions, and lesions previously treated with local therapy. First, osteolytic bone lesions or mixed lytic-blastic lesions with a measurable soft tissue component ≥10 mm could be considered as TL. However, osteoblastic bone lesions represent Non-TL. Second, cystic metastatic lesions ≥10 mm could be considered as TL. However, if noncystic TL are present in the same patient, these should be preferred. Finally, lesions with prior local treatment, e.g. radiation therapy or biopsy, should usually not be considered as target lesions unless there has been demonstrated clear tumor progression afterwards.

### Follow-up

Regular follow-up response assessment every 6–12 weeks is recommended for iRECIST. During iRECIST follow-up monitoring, in line with RECIST 1.1, all TL defined at baseline must be quantitatively re-measured and all Non-TL must be qualitatively re-evaluated (Fig. 2). The measurement of the maximum diameter of the TL at the new follow-up is independent of the previous direction of the measurement within the lesion or slice position, but always in identical slice orientation. In case a target lesion is reported as too small to measure but still visible, a default value of 5 mm could be used. In the rare case if a target lesion splits into two separate lesions, the separate measurements of the lesions should be added together for the target lesion sum. In case target lesions coalesce and are radiologically no longer separable, the maximum longest diameter for the coalesced lesion should be provided and the other lesion should be noted with 0 mm. Lymph node metastases are handled specifically. Even under a highly effective treatment in most cases they will never fully disappear and will only shrink to their physiological size. Lymph nodes are considered as tumor free once their SAD is < 10 mm, but the measurements should be recorded in all subsequent follow-ups in order not to overstate progression in case of a minor increase in size, e.g. from 9 mm to 11 mm. This means that when lymph node metastases are TL, the tumor burden will mostly not become ‘zero’ even in the case of a complete response. Please notice that a TL defined at baseline assessment always remains a TL, even if it shows a size reduction to less than 10 mm. Similarly, Non-TL yielding a size increase of more than 10 mm at follow-up remains a Non-TL but could qualify for ‘unequivocal progression’ in case of an overall level of substantial worsening in non-target disease.

With regards to the measurable TL, the proportional change of the sum of the target lesions can be calculated with the formula: Change in [%] = ((∑Follow-up - ∑Baseline/ ∑Nadir)/ ∑Baseline/ ∑Nadir) * 100. Taking as reference the smallest target sum in the study, so called Nadir, which could be the baseline target sum if that is the smallest sum in the study.

Non-TL are assessed qualitatively, i.e. visually, as either ‘present’, ‘disappeared’ or ‘unequivocal progression’. When considering determining an ‘unequivocal progression’ of Non-TL, the total tumor load should always be taken into account in proportion and carefully weighed, as this would necessarily imply classification of ‘progressive disease’, even if all other lesions have responded strongly or even completely. In case of doubt, the responsible oncologist should be consulted.

In contrast to RECIST 1.1, where new tumor lesions are considered qualitatively and directly denote ‘progressive disease‘ and end of study, within iRECIST, they are differentiated into new measurable and non-measurable lesions. Although new tumor lesions within iRECIST will also be classified as tumor progression, this progression initially counts as an ‘unconfirmed progressive disease’ (iUPD) which could be re-assessed in a dedicated earlier follow-up after 4-8 weeks. For classification as new measurable or non-measurable tumor lesions, criteria applied are the same as for the baseline examination with a maximum of 5 measurable new target lesions per patient and 2 per organ, respectively, which are measured as a separate group at the time of the first occurrence while the sum product of all new measurable TL is determined. The new non-measurable lesions are documented qualitatively similarly to the Non-TL at baseline. Tumor lesions diagnosed for the first time in a previously unexamined body region are also classified as ‘new lesions’ in line with RECIST 1.1. The rationale behind this procedure is that the extension of imaging to a previously unexamined region, which leads to the detection of new tumor lesions, is usually triggered by the occurrence of new clinical symptoms.

In case of a new unclear lesion, e.g. because of its small size, this lesion should be preferably noted as a ‘finding’, therapy should be continued, and follow-up evaluation could clarify if it represents truly new disease. If repeat examination confirms a new tumor lesion, then progression should be declared using the date of the initial scan when the lesion was first detected.

### Responses to therapy

The overall response according to iRECIST results from the combination of changes in TL and Non-TL, as well as the possible detection and change of new measurable and non-measurable tumor lesions. The objective response in the context of immunotherapy (with the prefix ‘i’ for immune-related) is differentiated into:

- Complete Response (iCR), which describes the complete disappearance of TL and Non-TL. All lymph nodes must be non-pathological in size (< 10 mm in SAD).
- Partial Response (iPR), which occurs when the tumor load of the TL is reduced by ≤30% compared to the baseline, or in the case of complete remission of the TL, when one or more Non-TL can still be distinguished.
- Stable Disease (iSD), which is to be determined if the criteria of iCR or iPR are not met and no tumor progression is present.

In case of a tumor progression, and in order to facilitate differentiation of true tumor progression from pseudoprogression in clinically stable patients, iRECIST proposes to determine first:

- unconfirmed Progressive Disease (iUPD) due to an increase in the sum of all TL by at least ≥20% (but at least ≥5 mm) compared to the time point with the lowest TL sum (Nadir), or an unequivocal progression of Non-TL, or by the occurrence of new measurable and/or non-measurable tumor lesions.

This initially unconfirmed tumor progression might be confirmed by a subsequent follow-up where:

- confirmed Progressive Disease (iCPD) is present if further progress of the target sum (≥ 5 mm), or any further progress of the Non-TL, and/or progress of the new measurable and not measurable lesions either in number or in size (sum ≥5 mm).

In case of iUPD, the follow-up for re-evaluation and diagnosis of potential pseudoprogression should be carried out earlier after 4–8 weeks, in contrast to the regularly recommended time interval of 6–12 weeks. In case tumor progression is not confirmed and TL, Non-TL and new lesions remain unchanged, ‘iUPD’ status should be kept and subsequent follow-up should be performed according to the regular schedule, e.g. after 8, 16 and 24 weeks. Moreover, if the tumor burden decreases more than 20%, this should be considered iSD; if it decreases less than 30%, this should be considered iPR. If tumor lesions completely disappear, there is iCR even after iUPD.

However, in iRECIST it is clearly recommended to carefully consider the continuation of immunotherapy at the first stage of tumor progression (iUPD). This decision should be thoroughly discussed critically with both, patient and referring physicians and only be made in case of subjective stable tumor disease or clinically suspected pseudoprogression. New lesions in a potentially curative therapy approach could be biopsied in order to enable a more reliable differentiation of rare pseudoprogression from more frequent progressive disease and to be able to initiate an early modification of the tumor therapy before the patient may no longer tolerate it due to a physical deterioration. In the case that a biopsy is not technically feasible or only feasible with a significantly increased risk, the confirmation of the less probable delayed therapy response can be represented by a follow-up after 4–8 weeks in subjectively stable tumor patients during this period.

According to RECIST 1.1 the RECIST working group not believed that there was sufficient data available to recommend implementation of metabolic and/or functional imaging response parameter. Exception is the use of FDG-PET imaging as an adjunct to determination of progression if a positive FDG-PET at follow-up corresponds to a new site of disease confirmed by CT [21]. However, the actual literature does not support the non-invasive differentiation of true progression from pseudoprogression by PET/CT.

For iRECIST, the best overall response (iBOR) is the best timepoint response recorded from the start of immunotherapy until the end of study treatment. iUPD will not override a subsequent best overall response of iSD, iPR, or iCR.

## Conclusions

The new iRECIST criteria allow a standardized response evaluation within the framework of clinical trials, considering the relatively rare, but clinically significant possibility of pseudoprogression within the framework of modern oncological immunotherapies. For therapy decisions in the oncological routine, iRECIST should be used with caution but may offer a good option to systematically document therapy outcome.

## Data Availability

Not applicable.

## Abbreviations

iCPD: Confirmed progressive disease
iCR: Complete remission
iPR: Partial remission
iSD: Stable disease
iUPD: Unconfirmed progressive disease
Non-TL: Non target lesion
NSCLC: Non-small-cell lung cancer
PD-1: Programmed death 1
PD-L1: Programmed death ligand 1CTLA-4: cytotoxic T-lymphocyte antigen 4
RECIST: Response Evaluation Criteria In Solid Tumors
TL: Target lesion
WHO: World Health Organization
